# Environmental context related to hydrogen sulphide and regional variation in utilisation of ROCK inhibitors in Japan: a nationwide ecological analysis

**DOI:** 10.1038/s41598-026-57364-x

**Published:** 2026-06-08

**Authors:** Ryosuke Shinkai, Takahiro Amemiya, Takashi Tomita

**Affiliations:** 1 Department of Pharmaceutical Sciences, School of Pharmacy at Narita, International University of Health and Welfare, 4-3 Kozunomori, Narita, 286-8686 Japan; 2 Department of Pharmacy, International University of Health and Welfare Narita Hospital, 854 Hatakeda, Narita, 286-8520 Japan; 3 Faculty of Pharmaceutical Sciences, Teikyo Heisei University, 4-21-2 Nakano, Nakano-ku, Tokyo 164-8530 Japan

**Keywords:** Diseases, Environmental sciences

## Abstract

Regional variation in healthcare utilisation is widely documented, yet the contribution of natural environmental context to prescribing patterns remains poorly understood. Geological environments shape long-term background exposure to naturally occurring substances, but population-level evidence linking geological context to ophthalmic prescribing is limited. Experimental studies have suggested that hydrogen sulphide (H_2_S) may interact with the RhoA/Rho kinase (ROCK) signalling pathway, which is the pharmacological target of ROCK inhibitor medications used for glaucoma. We conducted a nationwide ecological cross-sectional study using prefecture-level data from all 47 prefectures of Japan. Annual per-capita prescription volumes of ripasudil ophthalmic solution were obtained from the National Database of Health Insurance Claims, and geological context was quantified using the volcanic rock area ratio (VRAR) as a geological background indicator reflecting long-term environmental context, including potential exposure to H_2_S, rather than a direct measure of exposure. Associations were analysed using locally estimated scatterplot smoothing (LOESS), multiple linear regression, and random forest modelling with adjustment for demographic and healthcare-related covariates. Ophthalmic prescribing patterns showed marked regional variation across Japan, and analyses identified a consistent non-linear association between geological context and per-capita ROCK inhibitor prescriptions, with prescription levels peaking in prefectures with intermediate VRARs. Multivariable linear regression showed limited explanatory power, whereas random forest models suggested that geological context was among the most influential predictors among the examined variables. These findings suggest that regional geological context may represent a previously underexplored structural factor associated with variation in ophthalmic prescribing patterns, highlighting a potential intersection between environmental context and population-level pharmacological utilisation.

**Supplementary Information:**

The online version contains supplementary material available at 10.1038/s41598-026-57364-x.

## Introduction

Regional variation in healthcare utilisation is widely observed across countries and therapeutic areas and is recognised as an important topic in epidemiology and health services research^1–3^. This type of variation is not merely descriptive but has important implications for equity, efficiency, and resource allocation within health systems, as unexplained geographic heterogeneity may reflect contextual influences operating beyond individual-level risk factors. Differences in demographic structure, healthcare access, and provider distribution are commonly invoked to explain regional prescribing patterns; however, these factors often fail to fully account for observed variation. Therefore, identifying additional contextual determinants that shape population-level healthcare utilisation remains an important challenge in community and public health research.

Environmental context has increasingly been recognised as a contributor to population health^4^, yet its role in shaping patterns of healthcare utilisation has received comparatively limited attention. In particular, naturally occurring geological factors represent stable, long-term contextual exposures that differ systematically across regions but are rarely incorporated into pharmacoepidemiological analyses. Unlike short-term environmental fluctuations or individual-level behaviours, geological environments define persistent background conditions that may indirectly influence healthcare demand, prescribing behaviour, and treatment patterns at the population level.

H_2_S is a widely distributed trace gas in the natural environment, released primarily through volcanic and geothermal activity^5^. Although historically recognised for its toxicity at high concentrations, H_2_S is now understood to function as an endogenous gasotransmitter—alongside nitric oxide and carbon monoxide—with diverse physiological roles in mammals^6^. Endogenously, H_2_S is synthesised by cystathionine β-synthase, cystathionine γ-lyase, and 3-mercaptopyruvate sulphurtransferase and has been implicated in the regulation of vascular tone, oxidative stress, and inflammatory responses^7–9^. Experimental studies have also suggested that H_2_S may interact with the ROCK signalling pathway, a key regulator of cytoskeletal dynamics and smooth muscle contraction^10,11^. Pharmacological inhibition of this pathway underlies the therapeutic mechanism of antiglaucoma agents, including ripasudil and netarsudil^12–14^. Rather than implying a direct pharmacological substitution, environmental factors related to H_2_S may be conceptualised as part of a broader environmental context that is associated with variation in population-level treatment demand and prescribing behaviour.

Because ROCK inhibitors target this signalling pathway, environmental factors related to ROCK-associated signalling may be associated with variation in pharmacological utilisation patterns at the population level, although this relationship has not been established in epidemiological settings. This possibility has not yet been explored epidemiologically, and therefore raises the question of whether regional geological context, reflecting long-term environmental conditions including factors related to H_2_S[15], is associated with variation in the utilisation of glaucoma medications.

Geochemical processes associated with volcanic regions release trace amounts of H_2_S through soil, groundwater, and geothermal systems. The areal proportion of volcanic rock represents a stable geological characteristic and can serve as a geological indicator reflecting long-term environmental context, which may include factors related to sulphur-containing gases, rather than reflecting short-term or localised environmental conditions. Volcanic rock distribution is closely linked to geothermal activity and sulphur emission intensity and has been framed within the global geodynamic sulphur cycle^16^. In Japan, volcanic geology is strongly associated with regional patterns of geothermal fluid circulation and gas emissions, including H_2_S^17,18^, and global analyses have identified volcanic regions as hotspots of sulphuric gas release^19^.

Despite these established links between geology and environmental H_2_S, the potential influence of such long-term environmental context on healthcare utilisation has not been systematically examined. In particular, no previous study has investigated whether naturally occurring geological factors are associated with regional variation in the utilisation of pharmaceuticals targeting signalling pathways that are also influenced by environmental gases. This gap is notable given accumulating evidence that contextual environmental factors may shape healthcare utilisation patterns beyond conventional demographic and healthcare system determinants^1–4^.

Against this background, the present study was designed as a nationwide, proof-of-concept ecological analysis to explore whether regional geological context is associated with variation in the utilisation of ROCK inhibitors in Japan. Using the VRAR as a geological background indicator reflecting long-term environmental context, which may include factors related to H_2_S, rather than directly representing exposure levels, we analysed prefecture-level differences in per-capita prescriptions of ripasudil across all 47 prefectures as an indicator of community-level healthcare demand. We aimed to explore whether geological context may be associated with non-linear variation in the utilisation of ROCK inhibitors, rather than assuming a simple monotonic relationship. This study is designed as a hypothesis-generating ecological analysis and does not aim to establish causal relationships at the individual level. This study focuses on identifying population-level patterns rather than making causal inferences.

## Materials and methods

### Study design

This study was designed to characterise regional prescribing patterns of ROCK inhibitors in Japan at the prefectural level and to explore potential non-linear associations between VRARs and prescription volume. The analysis was descriptive and exploratory in nature and was not intended to estimate causal effects or individual-level associations.

### ROCK inhibitor prescription data

Annual prescription counts of ripasudil ophthalmic solution (branded as Glanatec)—the only ROCK inhibitor approved in Japan in 2022—were obtained from the National Database of Health Insurance Claims and Specific Health Checkups of Japan (NDB Open Data), which is maintained by the Ministry of Health, Labour and Welfare^20,21^. The NDB covers more than 95% of the Japanese population.

Prefectural population data were obtained from the Statistics Bureau of Japan for 2022 and were used to normalise prescription counts to per-capita values^22^. Population size was not included as an independent variable in subsequent multivariate analyses to avoid statistical dependencies, as per-capita normalisation already accounts for population differences across prefectures.

### Geological data

Geological information was derived from the Seamless Digital Geological Map of Japan (Version 2) provided by the Geological Survey of Japan^23^. VRARs were calculated in QGIS^24^ following these steps:

1. Extraction of volcanic rock polygons based on the *symbol* attribute
2. Spatial clipping using prefectural administrative boundaries
3. Calculation of volcanic rock area (km^2^)
4. Normalisation to total land area to obtain the VRAR (%)

VRAR was selected as a geological background indicator because it represents a stable, regionally defined geological characteristic that can be consistently quantified across all prefectures, enabling nationwide comparative analysis in the absence of direct exposure measurements.

The VRAR ranged from 2.77 to 88.76% across prefectures. This range was divided into tertiles defined as follows:

- T1 (low): 2.77–23.75%
- T2 (middle): 23.76–50.98%
- T3 (high): 50.99–88.76%

### Supplementary covariate data

Prefectural older-age proportion (percentage of residents aged ≥ 65 years)^25^, spatial clinic density^26^, and annual daylight duration^27^ data for 2022 were obtained from publicly available national datasets.

Spatial ophthalmologist density data were retrieved from datasets provided by the Japan Ophthalmologists Association^28^. These variables were included as covariates to account for differences in healthcare infrastructure, demographic structure, and baseline environmental context. Daylight duration was included as an environmental proxy potentially associated with circadian regulation, which may influence physiological parameters such as intraocular pressure and healthcare utilisation.

### Mapping

Spatial distributions were visualised using choropleth maps with ten colour categories (deciles), generated in QGIS (version 3.28)^24^.

### Statistical analysis

#### Visualisation of non-linear patterns (LOESS)

The relationship between VRAR and per-capita ROCK inhibitor prescriptions was examined using locally estimated scatterplot smoothing (LOESS) regression with a smoothing parameter of 0.6, selected to balance smoothing and overfitting, as commonly applied in exploratory ecological analyses[29]. To assess the robustness of the LOESS smoothing, sensitivity analyses were conducted using multiple smoothing parameters (frac = 0.4, 0.6, and 0.8), and the overall curve shape remained consistent across smoothing parameters (Supplementary Fig. 2).

#### Random forest analysis

To explore potential non-linear associations and interaction effects that cannot be captured by linear models, a random forest regression model was applied. This analysis was conducted as an exploratory approach rather than for predictive optimisation. The model included VRARs, older-age proportion, clinic density, ophthalmologist density, and daylight duration as explanatory variables. Models were fitted using commonly applied default hyperparameters (as implemented in standard R packages) to minimise the risk of overfitting given the limited sample size. The number of candidate variables considered at each split was set to one-third of the total predictors, and the minimum terminal node size was set to five.

The number of trees was selected based on model stability assessed using the out-of-bag (OOB) error.

The OOB error decreased rapidly in the early phase and reached a near-stable plateau thereafter (Fig. 3C). A larger ensemble of 400 trees was adopted to ensure robustness and stable estimation of variable effects, particularly for permutation-based variable importance measures. Variable importance was assessed based on the mean decrease in accuracy (permutation importance).

Model performance was assessed using out-of-bag (OOB) predictions.

The OOB R^2^ was calculated to evaluate the proportion of variance explained by the model, and the root mean squared error (RMSE) was used to quantify prediction error.

These metrics provide an internal validation of model performance without requiring explicit data splitting. The influence of VRAR on model predictions was visualised using individual conditional expectation (ICE) curves and partial dependence plots (PDPs), and all model-derived outputs (variable importance, PDPs, ICE curves, and performance metrics) were obtained from the same model specification.

#### Multiple linear regression

To evaluate possible linear associations as a baseline model for comparison with non-linear approaches, a multiple linear regression model was constructed with per-capita prescription volume as the dependent variable. Covariates included VRAR, older-age proportion, clinic density, ophthalmologist density, and daylight duration. Model assumptions were evaluated using residual diagnostics, and multicollinearity was assessed using variance inflation factors, all of which were within acceptable ranges.

#### Tertile-based comparison

Differences in prescription volumes across volcanic rock tertiles (T1–T3) were compared using the Kruskal–Wallis test. Cohen’s d was calculated as a descriptive measure of effect size without implying statistical significance. Boxplots overlaid with data points were generated to visually compare differences among tertiles.

### Software

Statistical analyses, including LOESS regression, random forest analysis, multiple linear regression, and tertile-based comparisons, were conducted using R (version 4.3.0)^30^, and geospatial analyses were performed using QGIS (version 3.28)^24^. Statistical significance was defined as a two-tailed *p*-value < 0.05.

### Ethical considerations

This study used publicly available, fully anonymised aggregate data. No personal information was included; therefore, ethical approval and informed consent were not required.

## Results

### Geographic distribution

**Fig. 1:**
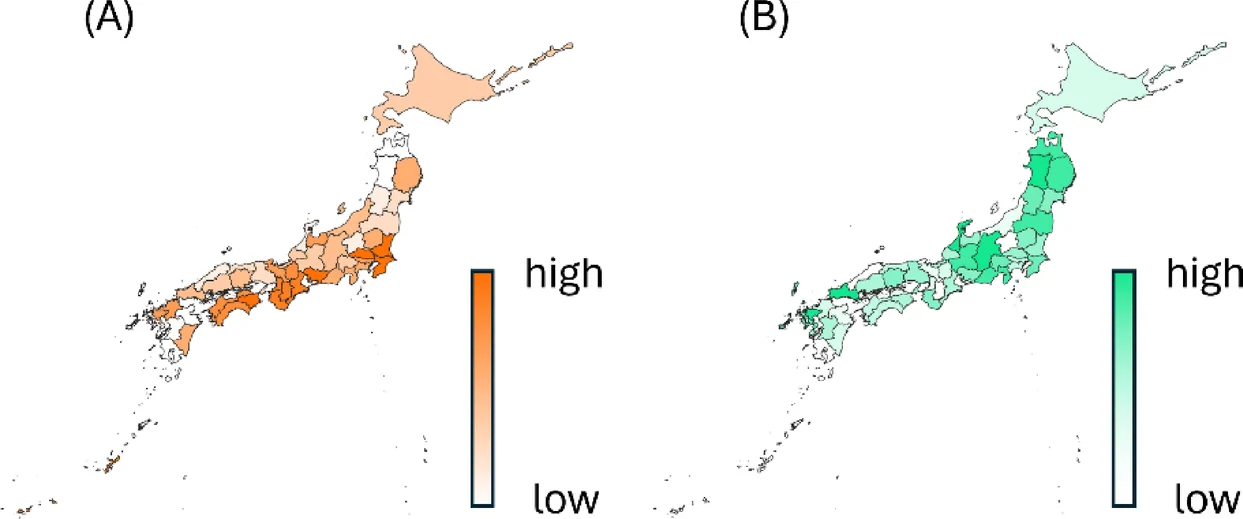
Geographic distribution of volcanic rock area ratio (VRAR) and ROCK inhibitor prescription volume across Japan. Choropleth maps showing (**A**) VRAR and (**B**) per-capita ROCK inhibitor prescriptions across Japan’s 47 prefectures. Both maps use ten colour-graded categories (deciles), with darker colours indicating higher values. The corresponding value ranges for each category are provided in Supplementary Table 1. The maps illustrate marked regional heterogeneity and the absence of a simple monotonic geographical pattern between VRAR and prescription volume. These maps are intended for descriptive visualisation and not for direct quantitative comparison between panels.

VRAR distributions showed a pronounced north–south gradient across Japan, with particularly high values observed in Hokkaido and Kyushu prefectures (Fig. 1A). In contrast, per-capita prescriptions of ripasudil exhibited a heterogeneous spatial pattern, with several low-use areas distributed across the country (Fig. 1B). No clear monotonic geographical trend was observed. This spatial heterogeneity was consistent with the scattered distribution of raw data points. Choropleth maps were used for qualitative visualisation and were not used for subsequent statistical inference. A descriptive geographic overview of major volcanic regions, representative volcanoes, and major urban centers across the Japanese archipelago, illustrating the spatial coexistence of volcanic activity and densely populated areas in Japan (Supplementary Fig. 1), was included to provide geographic context and was not directly used in statistical analyses.

### Non-linear association between VRARs and ROCK inhibitor prescriptions

**Fig. 2:**
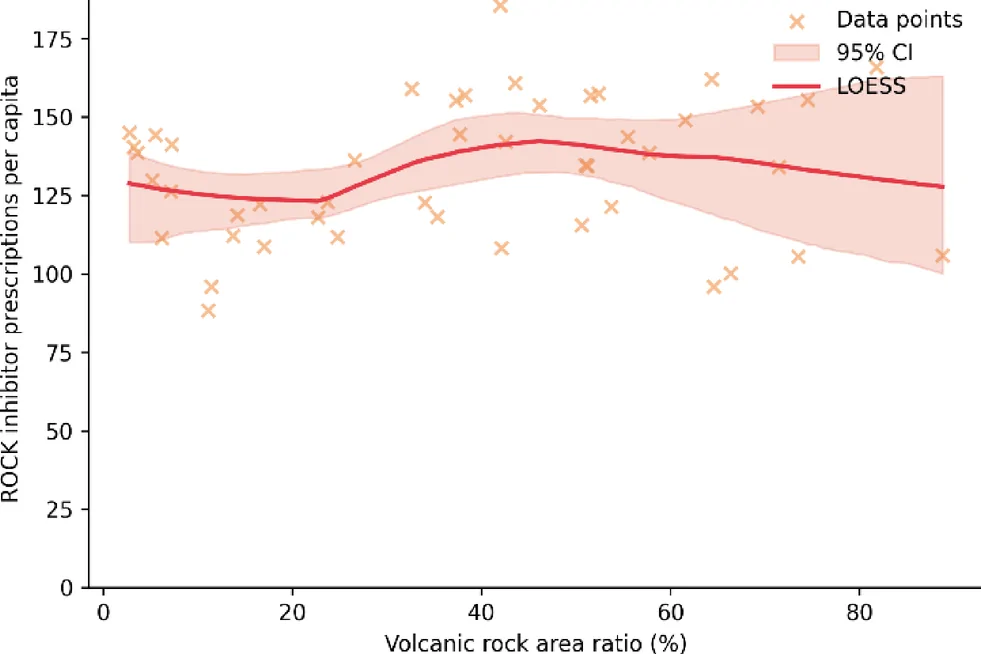
Association between volcanic rock area ratio (VRAR) and ROCK inhibitor prescriptions per capita. A locally weighted regression (LOESS) curve is shown with 95% confidence intervals. The analysis was based on prefecture-level data (n = 47). The smoothing parameter was set to frac = 0.6. Wider confidence intervals observed in higher VRAR ranges likely reflect increased variability in these regions, particularly given the limited number of observations.

LOESS regression indicated a non-linear relationship between VRAR and per-capita ROCK inhibitor prescriptions (Fig. 2). The smoothed curve was consistent with the distribution of raw data points, suggesting that the observed pattern reflected the overall structure of the data rather than sparsely distributed regional values. Prescription levels tended to be highest in prefectures with VRARs of approximately 30–50%, whereas both lower and higher ratios were associated with lower prescription volumes. This non-linear pattern remained consistent across sensitivity analyses using alternative LOESS smoothing parameters (Supplementary Fig. 2), which demonstrates the robustness of the observed non-linear association across different model specifications.

### Random forest analysis

**Fig. 3:**
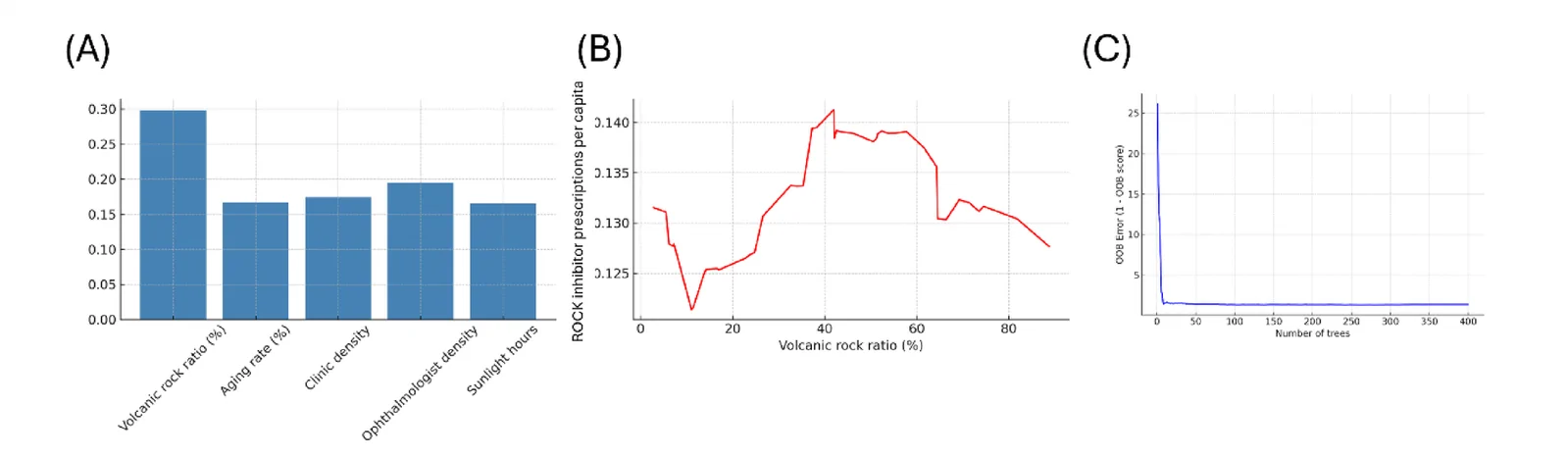
Random forest–based analysis of factors associated with ROCK inhibitor prescriptions per capita. (**A**) Variable importance from random forest analysis, expressed as relative feature importance derived from the random forest model and normalised to sum to 1. Higher values indicate greater contribution of each predictor within the fitted model. The corresponding relative importance values were 0.302 (VRAR), 0.166 (ageing rate), 0.174 (clinic density), 0.191 (ophthalmologist density), and 0.167 (daylight hours). (**B**) Partial dependence plot showing the association between volcanic rock area ratio (VRAR) and ROCK inhibitor prescriptions per capita. (**C**) Out-of-bag (OOB) error curve demonstrating model stability and convergence. The partial dependence plot illustrates the adjusted marginal effect of VRAR while averaging over other variables. The OOB error curve shows rapid error reduction followed by a near-stable plateau, suggesting model stability.

Random forest regression was applied to explore potential non-linear relationships and variable interactions beyond linear modelling. VRAR showed the highest permutation importance (mean decrease in accuracy) among the examined predictors within this exploratory model (Fig. 3A).

The relative importance values were 0.302 for VRAR, 0.166 for ageing rate, 0.174 for clinic density, 0.191 for ophthalmologist density, and 0.167 for daylight hours. However, this ranking should be interpreted as model-specific and exploratory, rather than indicative of causal relationships or generalisable predictive dominance. This ranking reflects relative importance within the fitted model and does not imply superiority over other variables in a broader epidemiological context.

The partial dependence plot (PDP) showed a smooth, non-linear response pattern: prescription volume increased from lower values at low VRARs toward a broad maximum in the mid-range, followed by a slight decline and an approximate plateau at higher VRARs (Fig. 3B). Individual conditional expectation (ICE) curves demonstrated variability across prefectures; however, many curves showed an increase in prescription volume within the mid-range of VRARs, followed by a mild decrease or plateau at higher ratios (Supplementary Fig. 3).

The random forest model showed limited predictive performance (OOB R^2^ =  − 0.33; OOB MSE = 2499.5; OOB RMSE = 50), which reflects the multifactorial nature of the outcome and is consistent with the exploratory aim of identifying non-linear patterns rather than optimising prediction accuracy. These findings should therefore be interpreted as exploratory and model-specific rather than as evidence of causal relationships.

### Comparison of LOESS and partial dependence plot results for supplementary covariates

LOESS and PDP analyses of prescription-volume dependence on older-age proportion, clinic density, ophthalmologist density, and daylight duration did not reveal clear non-linear patterns (Supplementary Fig. 5). For older-age proportion and clinic density, LOESS and PDP showed broadly similar shapes with shallow curvature. Ophthalmologist density exhibited slight curvature in LOESS, whereas its PDP remained nearly linear. Daylight duration showed the lowest agreement between LOESS and PDP, with LOESS displaying slight curvature and PDP remaining almost linear.

### Multiple linear regression

In the adjusted multivariate regression model including older-age proportion, clinic density, ophthalmologist density, and daylight duration (Table 1), the standardised coefficient for VRAR was small and not statistically significant (β = 0.156). This finding indicates that the linear regression model did not identify a statistically significant association between VRAR and prescription volume and suggests that the relationship may not be adequately captured by a linear model. The coefficients for the other covariates were also small, and the model showed limited explanatory power (adjusted R^2^ = 0.137). All variance inflation factors were within acceptable ranges, indicating negligible multicollinearity. Model fit metrics were as follows: adjusted R^2^ = 0.137, Akaike Information Criterion = 427.73, and Bayesian Information Criterion = 438.83. The limited explanatory power reflects the multifactorial nature of healthcare utilisation at the population level.

**Table 1:** Multiple linear regression analysis of factors associated with ROCK inhibitor prescriptions per capita across 47 prefectures in Japan.

Variable	Coefficient (β)	Standard error	Standardized β	95% CI			*p*-value
Intercept	83.11	52.17	–	– 22.24	–	188.47	0.119
Volcanic rock area ratio (%)	0.141	0.164	0.156	– 0.191	–	0.473	0.397
Older-age proportion (%)	123.78	118.77	0.185	– 116.08	–	363.64	0.303
Clinic density (per 100,000 population)	– 0.270	0.423	– 0.149	– 1.124	–	0.584	0.527
Ophthalmologist density (per 100,000 population)	– 2.19	2.95	– 0.171	– 8.16	–	3.78	0.463
Annual daylight duration (hours/year)	0.023	0.019	0.207	–0.015	–	0.062	0.232
					Adjusted R^2^ = 0.137		

Independent variables are VRARs, older-age proportion, clinic density, board-certified ophthalmologist density, and annual daylight hours; the dependent variable is ROCK inhibitor prescriptions per capita. Coefficients (β) and standard errors are presented in the original scale, whereas standardized β values represent effect sizes after Z-transformation of all variables. Ninety-five percent confidence intervals (95% CI) are shown for each parameter. The intercepts are unstandardized and included for completeness.

### Tertile analysis

In the tertile-based analysis, the middle tertile (T2) showed the highest prescription volumes (Supplementary Fig. 4). The Kruskal–Wallis test did not reach conventional statistical significance among VRAR tertiles (H = 5.01, *p* = 0.0818). Pairwise effect sizes (Cohen’s d) for the low–middle, middle–high, and low–high tertile comparisons were − 0.89, 0.18, and − 0.66, respectively.

Supplementary figures are referenced alongside the main results to provide additional detail and support interpretation of the observed patterns. This pattern, although not statistically significant, was directionally consistent with the non-linear trends observed in LOESS and random forest analyses.

## Discussion

To our knowledge, this study represents the first nationwide ecological analysis in Japan examining the association between a geological background indicator reflecting environmental context, including potential exposure to H_2_S, and regional utilisation patterns of ROCK inhibitors. By integrating multiple analytical approaches, including LOESS regression and random forest–based modelling, we identified a consistent non-linear prescribing pattern, characterised by a mid-range peak in utilisation rather than a monotonic trend. The consistency of this non-linear pattern across both LOESS and PDP analyses further supports the robustness of the observed association. On a regional scale, geochemical processes associated with volcanic rocks release trace amounts of H_2_S over long time periods^17,18^, resulting in geographically heterogeneous environmental contexts across the country, which may include factors related to sulphur-containing gases.

Using LOESS regression, a random forest (variable importances, ICE curves, and a PDP), and tertile-based comparisons, this study identified a consistent non-linear pattern in which ROCK inhibitor prescriptions peaked in prefectures with a moderate VRAR (approximately 30–50%). This pattern aligns with the scattered distribution of raw data points and suggests an underlying structure that cannot be captured by simple linear models. The linear regression model (Table 1) showed limited explanatory power of the tested covariates (adjusted R^2^ = 0.137), indicating that the phenomenon is not adequately described by linear associations. Accordingly, no monotonic trend in ROCK inhibitor prescriptions was observed, and the prominent mid-range peak detected by LOESS regression (Fig. 2) represents a notable population-level pattern warranting further consideration.

Although glaucoma prevalence increases with age^31,32^, age-related prescribing patterns may have weakened in recent years owing to the increasing availability of surgical treatment options. Moreover, ROCK inhibitors are typically administered as adjunctive therapies in cases where prostaglandin analogues are insufficient^33^, suggesting that regional prescription variation is likely influenced by complex and multifactorial factors beyond demographic structure alone.

Machine-learning analyses provided complementary insights. Random forest analysis (Fig. 3A) ranked VRAR as the highest among the examined predictors, and the PDP (Fig. 3B) reproduced a non-linear relationship characterised by a mid-range peak followed by a gradual decline and plateau. ICE curves (Supplementary Fig. 3), which illustrate prefecture-level variability in the response patterns, showed similar shapes across many prefectures, indicating broadly consistent non-linear patterns at the individual-prefecture level.

The OOB error decreased rapidly and reached a stable plateau, suggesting technical stability of the fitted model and supporting the chosen ensemble size, while remaining consistent with the exploratory nature of the analysis (Fig. 3C). Tertile comparisons (Supplementary Fig. 4) demonstrated that the highest prescription levels were observed in the middle group (T2), and the Cohen’s d values were consistent with this pattern. By contrast, older-age proportion, clinic density, ophthalmologist density, and daylight duration showed only slight curvature in both LOESS and PDP analyses (Supplementary Fig. 5), and none reproduced the distinct central peak observed for VRAR. Taken together, these analytical layers reinforce the internal coherence and cross-method consistency of the findings.

From an interpretative perspective, the observed non-linear association between VRAR and ROCK inhibitors prescription volumes may be interpreted in the context of existing physiological and experimental evidence. However, no direct biological measurements were performed in this study. H_2_S is naturally present in volcanic environments^34^ and is endogenously produced by cystathionine β-synthase, cystathionine γ-lyase, and 3-mercaptopyruvate sulphurtransferase as a gasotransmitter^35^. Experimental studies have suggested that H_2_S may interact with the RhoA/ROCK signalling pathway through S-sulfhydration and Ca2⁺-dependent mechanisms^11^. Rather than implying a direct causal mechanism, these findings provide a plausible biological context within which the population-level patterns observed in this study may be interpreted. These interpretations remain speculative and are intended solely to provide a conceptual framework for future investigation. One possible and speculative interpretation is that chronic environmental exposure to H_2_S may be related to variation in the broader biological context relevant to ROCK signalling. Such variation may be reflected in differences in pharmacological utilisation patterns at the population level. In Japan, environmental hydrogen sulphide exposure has been reported in association with geothermal and hot spring activity, although its spatial and temporal variability remains substantial^36,37^.

More broadly, these findings may be interpreted within the emerging concept of environmental modulation of pharmacological signalling pathways. H_2_S is known to regulate vascular and smooth muscle signalling through post-translational modifications such as protein sulfhydration. Because ROCK inhibitors target the same signalling cascade, chronic low-level environmental exposure may be associated with variation in the broader environmental context relevant to treatment patterns, which may be reflected in differences in pharmacological utilisation patterns at the population level.

Clinical studies in Japanese patients have reported that the intraocular-pressure-lowering effect of ROCK inhibitors does not increase proportionately at higher concentrations^38^, suggesting that additional ROCK inhibition may not translate into strictly dose-dependent clinical benefit. When viewed alongside previously reported non-linear biological responses to H_2_S exposure —protective at low concentrations, enhanced biological activity at moderate levels, and toxic at high concentrations^20,39^—these observations are consistent with the non-linear prescribing pattern identified in the present analysis. Such a mid-range peak is consistent with the well-described non-linear biological responses of H_2_S, in which low concentrations exert regulatory physiological effects whereas higher concentrations may induce cellular stress responses. Importantly, these interpretations are offered as hypothesis-generating perspectives rather than evidence of direct physiological causation. Several alternative explanations should be considered when interpreting the observed non-linear pattern. Regional differences in healthcare accessibility, ophthalmologist distribution, screening practices, and prescribing preferences may substantially influence prescription volume. In addition, areas with higher VRARs are often rural or geographically distinct, where healthcare access and disease detection rates may differ from urban regions. These contextual factors may partially account for the observed distribution, independent of environmental influences. One important unmeasured confounder is regional variation in glaucoma prevalence, which may directly influence prescription volume independent of environmental context. If glaucoma prevalence is higher in regions with greater VRAR, the observed association may reflect differences in disease burden rather than variation in treatment response or environmental effects.

In addition, prescription patterns may be influenced by regional socioeconomic conditions, including income level, patient education, healthcare accessibility, and local clinical practice patterns. Differences in health insurance reimbursement systems and regional healthcare infrastructure may also contribute to variation in prescribing behaviour. In addition, other volcano-related environmental factors, including additional gases and mineral components, may contribute to the observed patterns and cannot be disentangled within the current ecological framework.

These factors were not included in the present analysis and may partially or substantially account for the observed regional variation. At present, clinical evidence linking environmental H_2_S exposure to ocular physiological parameters, such as intraocular pressure or microcirculation, remains limited. Such regional variation in prescribing patterns has also been reported in previous pharmacoepidemiological studies, reflecting differences in healthcare accessibility, clinical practice, and population characteristics^40,41^.

This study has several limitations. In addition, because geological features do not align strictly with administrative boundaries, the use of prefectural-level aggregation may introduce spatial heterogeneity that cannot be fully captured in this ecological framework. First, as an ecological analysis conducted at the prefectural level, causal inference at the individual level is not possible^4,42^. Second, VRAR reflects regional geological characteristics but does not capture fine-scale spatial heterogeneity in environmental H_2_S exposure, such as local hot spring density, fumarole distribution, or seasonal emission patterns. Importantly, VRAR should not be interpreted as a direct measure of hydrogen sulphide exposure, but rather as a broad geological background indicator. Previous ecological studies have highlighted that proxy exposure variables, while useful for hypothesis generation, are subject to measurement limitations and may not accurately reflect individual-level exposure^42,43^, thereby limiting causal interpretation. Third, despite adjustment for clinic density and ophthalmologist numbers, unmeasured confounding factors—such as healthcare access pathways, care-seeking behaviour, or socioeconomic conditions—may remain. In particular, additional factors such as regional socioeconomic conditions, variation in glaucoma prevalence, differences in the use of alternative antiglaucoma medications, and heterogeneity in clinical guideline implementation may contribute to regional prescribing patterns. These factors were not included in the present analysis due to the limitations of publicly available aggregated data. Therefore, the observed associations should be interpreted in the context of a multifactorial system in which multiple unmeasured determinants may influence prescribing variability. These unmeasured factors may substantially influence the observed associations and should be considered when interpreting the limited explanatory power of the regression model. Fourth, per-capita prescription indicators do not capture underlying glaucoma prevalence or treatment stage, which may influence regional prescription profiles. Fifth, direct measurement of environmental H_2_S exposure was not available. Because H_2_S is highly reactive and readily converts to SOx and other sulphur compounds, accurate exposure assessment remains technically challenging, although recent advances in portable H_2_S sensor technologies may help address this limitation^44^.

Therefore, the findings should be interpreted as population-level associations rather than precise representations of local environmental exposure. Although prescription volume does not directly reflect disease severity, intraocular pressure control, or treatment efficacy, it captures real-world clinical decision-making and population-level treatment strategies. Despite these limitations, this study has notable strengths. It leverages nationwide data from all 47 prefectures in Japan and demonstrates consistency across multiple statistical and machine-learning frameworks. By framing geological context as one of several potential contributors to regional variation in healthcare utilisation, the findings provide a novel population health perspective that complements conventional demographic and healthcare system explanations. This interdisciplinary approach offers a foundation for future research examining links between environmental gas exposure and pharmacological responsiveness in ocular disease.

## Conclusion

This nationwide ecological analysis identified a non-linear association between geological context, as reflected by VRAR, and regional variation in ROCK inhibitor prescription patterns. These findings should be interpreted cautiously given the ecological design, the use of a proxy variable for environmental exposure, and the potential for unmeasured confounding.

Rather than providing definitive evidence, this study proposes a novel hypothesis that long-term environmental context may be associated with variation in pharmacological utilisation patterns at the population level. These findings are consistent with a hypothesis-generating framework and should be interpreted accordingly.

Future studies incorporating individual-level exposure assessment, biological markers, and finer spatial resolution are required to further evaluate and validate these observations.

## Supplementary Information

Below is the link to the electronic supplementary material.

Supplementary Material 1

## Data Availability

All data used in this study were obtained from publicly accessible governmental databases. Prescription data were obtained from the National Database of Health Insurance Claims (NDB), and geological data on VRARs were obtained from the Seamless Digital Geological Map provided by the National Institute of Advanced Industrial Science and Technology. No individual-level or personally identifiable information was included. Details of data sources and access links are provided in the Materials and Methods section.
