# Supplementary material for: Environmental context related to hydrogen sulphide and regional variation in utilisation of ROCK inhibitors in Japan: a nationwide ecological analysis
Source: Sci Rep. 2026 Jun 8;16:26091. doi: 10.1038/s41598-026-57364-x (PMC13490513; doi:10.1038/s41598-026-57364-x)
Supplement: Supplementary file 1 [file 41598_2026_57364_MOESM1_ESM.docx]

**Supplementary Figure 1. Geographic distribution of major volcanic regions, representative volcanoes, and major urban centers across Japan.**
The map was generated by the authors using QGIS version 3.28 (https://qgis.org). Volcanic regions derived from the Geological Survey of Japan, National Institute of Advanced Industrial Science and Technology, were overlaid on a prefectural map to provide geographic context regarding potential population exposure to volcanic environmental factors. Representative volcanoes and major urban centers are indicated for descriptive visualization of the spatial coexistence of volcanic activity and densely populated areas across the Japanese archipelago, including surrounding islands. This figure was included for geographic contextualization and was not directly used in statistical analyses.
Orange shaded areas indicate volcanic regions derived from the Geological Survey of Japan, while representative volcanoes and major urban centres are labelled for descriptive visualisation.

**Supplementary Figure 2. Sensitivity analysis of LOESS smoothing.**

LOESS curves were fitted using different smoothing parameters (frac = 0.4, 0.6, and 0.8). The overall non-linear pattern, characterised by a mid-range peak in prescription volume, was consistent and robust across different smoothing parameters.

**Supplementary Figure 3. Individual conditional expectation (ICE) curves and partial dependence plots (PDPs) for volcanic rock area ratio.**

Grey lines represent ICE trajectories for individual prefectures, showing the model-predicted ROCK inhibitor prescriptions per capita as the volcanic rock area ratio varies while other variables remain constant. The red line represents the PDP, illustrating the average marginal effect across all observations.

**Supplementary Figure 4. ROCK inhibitor prescriptions per capita across tertiles of volcanic rock area ratio.**

Prefectures were categorized into low, middle, and high tertiles based on volcanic rock area ratio.

A box-and-whisker plot is shown for each tertile, overlain with data points representing individual prefecture-level ROCK inhibitor prescriptions (blue—low, green—middle, red—high).
Pairwise effect sizes (Cohen’s d) are displayed in the upper right corner. A Kruskal–Wallis test indicated a marginal overall group difference (H = 5.01, p = 0.0818), supporting a non-linear pattern rather than a simple monotonic trend.

**Supplementary Figure 5.
LOESS and random forest–based visualisation of covariates associated with ROCK inhibitor prescriptions.**

LOESS curves and partial dependence plots (PDPs) for older-age proportion (A), clinic density (B), ophthalmologist density (C), and daylight duration (D) in relation to per-capita ROCK inhibitor prescriptions. The LOESS curves indicate nonparametric smoothed trends based on observed data, while the partial dependence plots (PDPs) show the effects of each explanatory variable estimated from a machine learning model with other variables held constant.

**Supplementary Table 1. Value ranges for decile-based categorisation used in Figure 1.**

Value ranges corresponding to each decile category are presented below.

| Volcanic rock area ratio (%) |  | ROCK inhibitor prescriptions per capita  (per 1,000 population) |
| --- | --- | --- |
| Decile 1: 2.774–5.925 |  | Decile 1: 88.321–105.789 |
| Decile 2: 5.925–11.915 |  | Decile 2: 105.789–111.806 |
| Decile 3: 11.915–21.564 |  | Decile 3: 111.806–118.634 |
| Decile 4: 21.564–33.182 |  | Decile 4: 118.634–124.245 |
| Decile 5: 33.182–38.239 |  | Decile 5: 124.245–134.677 |
| Decile 6: 38.239–45.113 |  | Decile 6: 134.677–140.930 |
| Decile 7: 45.113–51.721 |  | Decile 7: 140.930–144.639 |
| Decile 8: 51.721–60.814 |  | Decile 8: 144.639–154.957 |
| Decile 9: 60.814–70.116 |  | Decile 9: 154.957–158.066 |
| Decile 10: 70.116–88.764 |  | Decile 10: 158.066–185.641 |
